# Supplementary material for: Temperature-dependent development of the blow fly Chrysomya pinguis and its significance in estimating postmortem interval
Source: R Soc Open Sci. 2019 Sep 4;6(9):190003. doi: 10.1098/rsos.190003 (PMC6774990; doi:10.1098/rsos.190003)
Supplement: Supplementary Table 1 [file rsos190003supp1.docx]

Supplementary Table 1 The life stages of *Chrysomya pinguis* related to sample ages (hours)

| Age (hours) | 16℃ | 19℃ | 22℃ | 25℃ | 28℃ | 31℃ | 34℃ |
| --- | --- | --- | --- | --- | --- | --- | --- |
| 0-8 | E | E | E | E | E | E | E |
| 12-16 | E | E | E | E | E | L1: 40 | L1: 40 |
| 20 | E | E | E | E | L1: 40 | L1: 40 | L1: 40 |
| 24 | E | E | E | L1: 40 | L1: 40 | L1: 8, L2: 32 | L2: 40 |
| 28-32 | E | E | L1: 40 | L1: 40 | L1: 40 | L2: 40 | L2: 40 |
| 36 | E | L1: 40 | L1: 40 | L1: 40 | L1: 24, L2: 16 | L2: 40 | L2: 16, L3: 24 |
| 40 | E | L1: 40 | L1: 40 | L1: 32, L2: 8 | L2: 40 | L2: 32, L3: 8 | L3: 40 |
| 44 | E | L1: 40 | L1: 40 | L1: 32, L2: 8 | L2: 40 | L3: 40 | L3: 40 |
| 48 | E | L1: 40 | L1: 40 | L1: 7, L2: 32 | L2: 40 | L3: 40 | L3: 40 |
| 52 | L1: 40 | L1: 40 | L1: 40 | L2: 40 | L2: 40 | L3: 40 | L3: 40 |
| 56 | L1: 40 | L1: 40 | L1: 32, L2: 8 | L2: 40 | L2: 24, L3: 16 | L3: 40 | L3: 40 |
| 60 | L1: 40 | L1: 40 | L2: 40 | L2: 40 | L2: 16, L3: 24 | L3: 40 | L3: 40 |
| 64 | L1: 40 | L1: 40 | L2: 40 | L2: 40 | L3: 40 | L3: 40 | L3: 40 |
| 68 | L1: 40 | L1: 40 | L2: 40 | L2: 40 | L3: 40 | L3: 40 | L3: 8, W |
| 72 | L1: 40 | L1: 40 | L2: 40 | L2: 16, L3: 24 | L3: 40 | L3: 27, W | W |
| 76 | L1: 40 | L1: 40 | L2: 40 | L2: 8, L3: 32 | L3: 40 | L3: 16, W | W |
| 80 | L1: 40 | L1: 40 | L2: 40 | L3: 40 | L3: 40 | L3: 16, W | W |
| 84 | L1: 40 | L1: 37, L2: 1 | L2: 40 | L3: 40 | L3: 40 | W | W |
| 88 | L1: 40 | L2: 40 | L2: 40 | L3: 40 | L3: 40 | W | W |
| 92 | L1: 40 | L2: 40 | L2: 8, L3: 32 | L3: 40 | L3: 40 | W | W |
| 96 | L1: 40 | L2: 40 | L3: 40 | L3: 40 | L3: 10, W | W | P |
| 100-116 | L1: 40 | L2: 40 | L3: 40 | L3: 40 | W | W | P |
| 120 | L1: 34, L2: 5 | L2: 40 | L3: 40 | L3: 40 | W | W | P |
| 124 | L1: 26, L2: 14 | L2: 40, L3: 1 | L3: 40 | L3: 30, W | W | P | P |
| 128 | L2: 40 | L3: 39 | L3: 40 | L3: 20, W | P | P | P |
| 132 | L2: 40 | L3: 39 | L3: 40 | L3: 8, W | P | P | P |
| 136-144 | L2: 40 | L3: 40 | L3: 40 | W | P | P | P |
| 148 | L2: 40 | L3: 40 | L3: 32, W | W | P | P | P |
| 152 | L2: 40 | L3: 40 | L3: 30, W | W | P | P | P |
| 156 | L2: 40 | L3: 40 | L3: 18, W | W | P | P | P |
| 160 | L2: 40 | L3: 40 | W | W | P | P | P |
| 164-180 | L2: 40 | L3: 40 | W | P | P | P | P |
| 184 | L2: 40 | L3: 40 | W | P | P | P | A |
| 188-204 | L2: 40 | L3: 40 | W | P | P | P |  |
| 208 | L2: 33, L3: 7 | L3: 40 | P | P | P | A |  |
| 212 | L2: 32, L3: 8 | L3: 40 | P | P | P |  |  |
| 216-236 | L3: 40 | L3: 40 | P | P | P |  |  |
| 240 | L3: 40 | L3: 20, W | P | P | P |  |  |
| 244-248 | L3: 40 | W | P | P | P |  |  |
| 252 | L3: 40 | W | P | P | A |  |  |
| 256-292 | L3: 40 | W | P | P |  |  |  |
| 296-304 | L3: 40 | P | P | P |  |  |  |
| 308 | L3: 40 | P | P | A |  |  |  |
| 312-344 | L3: 40 | P | P |  |  |  |  |
| 348 | L3: 6, W | P | P |  |  |  |  |
| 352-376 | W | P | P |  |  |  |  |
| 380 | W | P | A |  |  |  |  |
| 384-420 | W | P |  |  |  |  |  |
| 424-530 | P | P |  |  |  |  |  |
| 534 | P | A |  |  |  |  |  |
| 538-806 | P |  |  |  |  |  |  |
| 810 | A |  |  |  |  |  |  |

E: Egg, L1: 1st instar, L2: 2nd instar, L3: 3rd instar, W: Wandering, P: Puparia, A: Adult
